# Evaluation of Interstitial Lung Disease Complications Caused by Biologic Agents Using a Spontaneous Adverse Drug Reaction Reporting Database

**DOI:** 10.1002/prp2.70063

**Published:** 2025-02-21

**Authors:** Ayu Minagi, Hideki Nawa, Mitsuhiro Goda, Takahiro Niimura, Koji Miyata, Hirofumi Hamano, Yoshito Zamami, Keisuke Ishizawa

**Affiliations:** ^1^ Department of Pharmacy, Faculty of Pharmacy Shujitsu University Okayama Japan; ^2^ Department of Pharmacy Okayama University Hospital Okayama Japan; ^3^ Department of Pharmacy Okayama Kyokuto Hospital Okayama Japan; ^4^ Department of Pharmacy Tokushima University Hospital Tokushima Japan; ^5^ Department of Clinical Pharmacology and Therapeutics Tokushima University Graduate School of Biomedical Sciences Tokushima Japan

**Keywords:** adverse drug reaction reporting systems, adverse event signal, biologic agent, drug‐related side effects and adverse reactions, interstitial, interstitial lung disease, lung diseases

## Abstract

Interstitial lung disease (ILD) is a clinically relevant adverse event associated with biologic agent use. However, the current incidence of ILD remains unclear as large‐scale risk assessments of biologic agents have not been conducted. The aim of this study was to clarify the association between biologic agent use and ILD development in clinical practice by detecting adverse event signals using a spontaneous adverse drug reaction database. The VigiBase database is used for spontaneous adverse event reporting. The analysis focused on nine biologics used to treat psoriasis, rheumatoid arthritis, and Crohn's disease. The safety of each biologic agent was evaluated using the information component signal detection method. There were 32,520,983 reports in VigiBase, of which 68,489 (0.21%) were for ILD. Signals were mainly detected for tumor necrosis factor‐α inhibitors when the information component for ILD caused by biologic agents was calculated. Comorbidity analysis in patients who developed ILD and analysis of the time from the start of treatment with each drug to ILD onset showed differences for each biologic agent. ILD is a serious adverse effect of biologic agents, and there are several cases in which a causal relationship with ILD development cannot be ruled out. The occurrence of interstitial ILD should be noted when using biologics, particularly TNF‐α inhibitors.

## Graphical abstract

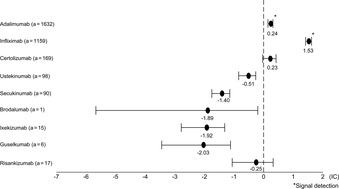

**Abbreviations**
ADR: adverse drug reaction
CI: confidence interval
IC: information component
IL: interleukin
ILD: interstitial lung disease
MedDRA: Medical Dictionary for Regulatory Activities
TNF‐α: tumor necrosis factor‐α
UMC: Uppsala Monitoring Centre
WHO: World Health Organization

## 1 Introduction

As the pathophysiology of refractory immune‐inflammatory diseases has been elucidated and the cytokines and molecules that play major roles in the establishment and maintenance of inflammation have been identified, drugs designed to target and inhibit these substances, commonly referred to as biologic agents, are being developed and increasingly used in the treatment of various diseases [1]. Biologic agents are chemically synthesized compounds/drugs made from materials derived from living organisms such as humans (often immunoglobulins or their genes) and created using the latest bioengineering techniques. Biologic agents have been developed for various rheumatic diseases, inflammatory bowel diseases such as Crohn's disease, and dermatological conditions such as psoriasis. They exhibit remarkable therapeutic effects, and their high clinical efficacy has attracted considerable attention [1]. Nevertheless, the inhibition of cytokines and other molecules targeted by these drugs may lead to undesirable adverse effects as these molecules play important roles in the complex network of the immune system [1]. In fact, several case reports on biologics have been published [2, 3, 4, 5, 6]. However, these adverse effects have not yet been thoroughly investigated or clarified. In particular, interstitial lung disease (ILD) has attracted clinical attention as an adverse event associated with biologic use. Cytokines, such as tumor necrosis factor‐α (TNF‐α), interleukin‐13 (IL‐13), IL‐6, and IL‐17, are associated with the development of ILD [7, 8]. However, it is unclear which cytokines act on which receptors to cause lung damage [7], and there is still no globally established method for selecting biologic agents [9].

Four cases of a causal relationship with ILD development have been reported for Toltz Subcutaneous Injection Autoinjectors, a biologic agent launched in November 2016; the opinions of expert committee members were also considered as domestic cases have accumulated. The Pharmaceuticals and Healthcare Bureau of the Ministry of Health, Labour and Welfare instructed the Federation of Pharmaceutical Manufacturers' Associations of Japan to revise the package inserts of drugs for which serious adverse reactions were identified in June 2021 [10]. In July 2021, the package insert for ixekizumab was revised to add ILD to “serious adverse reactions” to alert patients [11]. However, large‐scale risk assessments were not conducted for ixekizumab and other currently used biologic agents, and the incidence of ILD remains unclear.

In this study, we used VigiBase, a database of spontaneous adverse event reports published by the World Health Organization (WHO), which is useful for the analysis of post‐marketing adverse event reports. We aimed to elucidate the association between biologic agent use and ILD development. Specifically, we examined the occurrence of ILD attributable to various biologics used in clinical practice, the associated complications in patients who develop ILD, and the time elapsed between the administration of each biologic and onset of ILD. Through signal detection analysis in VigiBase, we sought to provide comprehensive insights into the correlation between biologic use and ILD onset.

## 2 Methods

### 2.1 Database

We collected spontaneous adverse event reports from VigiBase, a global database containing individual case reports of suspected adverse drug reactions (ADRs) from member countries of the WHO International Drug Surveillance Program since 1968. As anonymized data were used for analysis, obtaining patient consent was not necessary. The study complied with the “Ethical Code for Epidemiological Research” established by the Ministry of Health, Labour and Welfare [12, 13]. Furthermore, review committee approval was not required because the data in the database are anonymized [12, 13]. VigiBase consists of eight files: DEMO, ADR, DRUG, FOLLOWUP, IND, OUT, LINK, and SRCE. DEMO contains basic patient information such as sex, age, date of adverse event occurrence, and country of occurrence; ADR contains the name of the adverse event; DRUG contains information such as drug name, drug involvement, route of administration, and dosage; FOLLOWUP contains information on follow‐up; IND contains information on indication; OUT contains information on severity of the event; and LINK contains information such as Time To OnsetMin and Time To OnsetMax. In this study, LINK information was used to analyze the period from the start date of drug administration to the date of ADR. The analysis period for this study was between 1968 and 2022.

### 2.2 Analysis Target

The names used for the extraction of adverse events were the basic preferred terms searched under a narrow scope for ILD listed in the ICH Medical Dictionary for Regulatory Activities (MedDRA) Ver. 25.0J as standardized MedDRA queries (Table S1). Adalimumab, infliximab, certolizumab, ustekinumab, secukinumab, brodalumab, ixekizumab, guselkumab, and risankizumab, which are biologic agents used primarily in the treatment of psoriasis, rheumatoid arthritis, and Crohn's disease, were the drugs analyzed [9].

### 2.3 Analysis Method

NaviCat for SQLite (Premium Soft) ver. 15.0.29 was used to create the VigiBase database. Information component (IC) analysis, a signal detection method, was used to evaluate the safety of each biologic agent [14, 15]. IC is a central concept in the Bayesian confidence propagation neural network used at the Uppsala Monitoring Centre (UMC) of the WHO, which is designed to avoid inflation, particularly in instances with a limited number of reports [16, 17, 18]. The lower and upper limits of the 95% confidence interval (CI) were set as IC_025_ and IC_975_, respectively. The UMC method assumes that a signal is present when the lower limit of the calculated IC's 95% CI is positive and does not contain 0 [16].

$$\mathrm{IC}=\log_{2}\left[\frac{N_{\text{observed}}+0.5}{N_{\text{expected}}+0.5}\right],$$

$$\mathrm{IC}=\log_{2}\left[\frac{a+0.5}{\frac{\left(a+b\right)\left(a+c\right)}{a+b+c+d}+0.5}\right],$$

$${\mathrm{IC}}_{025}=\mathrm{IC}-3.3{\left(a+0.5\right)}^{-\frac{1}{2}}-2{\left(a+0.5\right)}^{-\frac{3}{2}},$$

$${\mathrm{IC}}_{975}=\mathrm{IC}+2.4{\left(a+0.5\right)}^{-\frac{1}{2}}-0.5{\left(a+0.5\right)}^{-\frac{3}{2}};$$

where IC indicates the information component; IC_025_ indicates the lower limit of the 95% confidence interval for the IC value was set at IC_025_; IC_975_ indicates the upper limit of the 95% confidence interval for the IC value was set at IC_975_; *a* is the number of cases of ILD caused by each biologic agent; *b* is the number of cases of adverse events other than ILD caused by each biologic agent; *c* is the number of cases of adverse events other than ILD caused by non‐biologic agents; *d* is the total number of cases with drugs other than biologic agents.

## 3 Results

**FIGURE 1:**
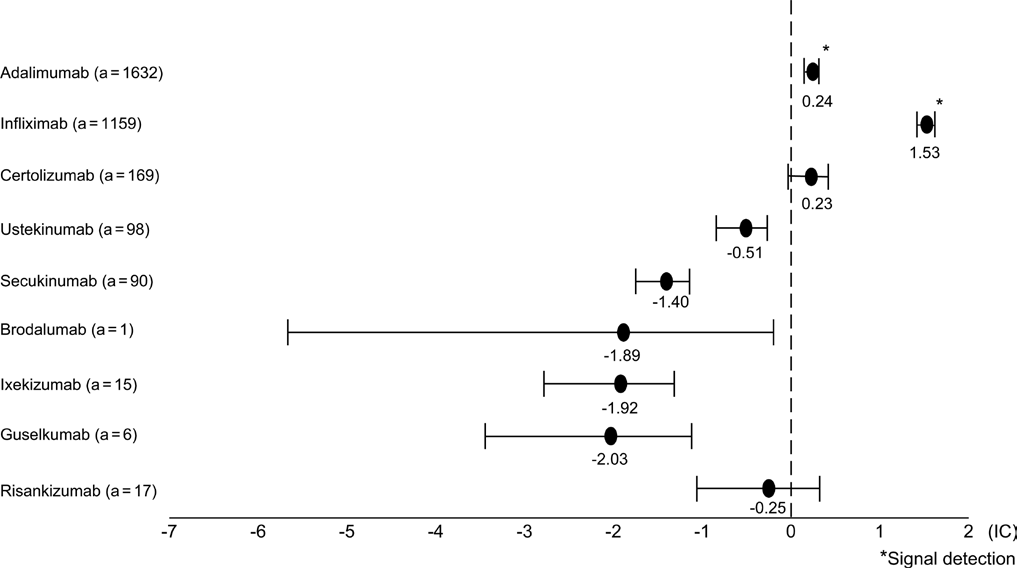
Information component (IC) (95% confidence interval: CI) for interstitial lung disease (ILD) onset for biologic agents. A signal was present when the lower limit of the 95% CI of the calculated IC was greater than 0. *a* = number of patients with ILD administered each biologic agent.

The total number of VigiBase reports was 32,520,983, with 68,489 (0.21%) ILD reports. The VigiBase IC (95% CI) for adalimumab, infliximab, certolizumab, ustekinumab, secukinumab, brodalumab, ixekizumab, guselkumab, and risankizumab was 0.24 (95% CI: 0.16–0.30), 1.53 (95% CI: 1.43–1.60), 0.23 (95% CI: −0.03 to 0.41), −0.51 (−0.84 to −0.27), −1.40 (−1.75 to −1.15), −1.89 (−5.67 to −0.20), −1.92 (−2.79 to −1.32), −2.03 (−3.44 to −1.12), and −0.25 (−1.07 to 0.32), respectively (Figure 1 and Table S2). Adalimumab and infliximab had a lower 95% CI limit of IC above 0, and a signal was detected.

As mentioned above, signals were mainly detected for TNF‐α inhibitors in association with the onset of ILD; therefore, further detailed analysis of TNF‐α inhibitors was conducted. The following adverse events upon TNF‐α inhibitor, adalimumab, infliximab, and certolizumab, administration were observed in patients who developed ILDs: adalimumab: edema (51.67%), tuberculosis (24.75%), and upper respiratory tract infection (9.82%); infliximab: abdominal distention (58.91%), acute respiratory failure (10.08%), and anorexia (8.91%); certolizumab: dyspnea (83.92%), hematochezia (8.93%), and unknown cause of death (3.57%) (Table 1).

**TABLE 1:** Adverse events following biologic agent administration in patients with interstitial lung disease.

Biologic agent	Preferred term	Number of reports	%
Adalimumab	Edema	263	51.67
	Tuberculosis	126	24.75
	Upper respiratory tract infection	50	9.82
	Squamous cell carcinoma	22	4.32
	Muscle strain	11	2.16
	Diarrhea	11	2.16
	Dyspnea	9	1.77
	Aortic aneurysm rupture	8	1.57
	Fatigue aggravated	3	0.59
	Adverse drug reaction	2	0.39
	Pneumonia	1	0.20
	Fever	1	0.20
	Lung injury	1	0.20
	Renal failure	1	0.20
		Total 509	Total 100.00
Infliximab	Abdominal distension	152	58.91
	Acute respiratory failure	26	10.08
	Anorexia	23	8.91
	Asthma	20	7.75
	Atrial fibrillation	14	5.43
	Cryptogenic organizing pneumonia	10	3.88
	Arthropathy	6	2.32
	Fever	4	1.55
	Myelodysplastic syndrome	1	0.39
	Respiratory tract infection	1	0.39
	Oral fungal infection	1	0.39
		Total 258	Total 100.00
Certolizumab	Dyspnea	47	83.92
	Hematochezia	5	8.93
	Unknown cause of death	2	3.57
	Stevens–Johnson syndrome	1	1.79
	Injection site pain	1	1.79
		Total 56	Total 100.00

Regarding the duration of reporting in VigiBase for ILD from the start of dosing, the longest median duration was for adalimumab among TNF‐α inhibitors, with a mean of 644.7 days and a median (interquartile range: 25%–75%) of 320.5 (117.1–821.9) days. Secukinumab had the longest median duration among the interleukin inhibitors, with a mean of 303.5 days and a median (interquartile range: 25%–75%) of 206.5 (94.5–424.1) days (Table 2).

**TABLE 2:** Medians of time‐to‐onset of interstitial lung disease (preferred terms) in VigiBase.

Drug	Number of reports	Average (days)	Median of time‐to‐onset
Adalimumab	514	644.7	320.5 (117.1–821.9)
Infliximab	407	529.5	127.0 (61.3–500.0)
Certolizumab	52	382.1	82.0 (51.8–316.7)
Ustekinumab	33	198.9	90.0 (31.0–235.0)
Secukinumab	46	303.5	206.5 (94.5–424.1)
Brodalumab	0	N/A	N/A
Ixekizumab	8	312.5	169.0 (68.3–415.5)
Guselkumab	3	252.7	123.0 (111.0–329.5)
Risankizumab	0	N/A	N/A

Abbreviation: N/A, not applicable.

## 4 Discussion

This study was initiated in response to the addition of ILD as a serious adverse effect in the package insert of ixekizumab. The study focused on ILD complications as adverse events of TNF‐α inhibitors and analyzed potential complications of ILD associated with other biologic agents. The biologic agents used in the study were classified based on their mechanisms of action: adalimumab, infliximab, and certolizumab are TNF‐α inhibitors, and ustekinumab, secukinumab, brodalumab, ixekizumab, guselkumab, and risankizumab are interleukin inhibitors. The Japanese package inserts list ILDs as serious adverse reactions for adalimumab, infliximab, certolizumab, ustekinumab, and ixekizumab [11, 19, 20, 21, 22]. The US Food and Drug Administration package inserts list ILDs as serious adverse reactions for adalimumab, infliximab, and ustekinumab [23, 24, 25]. The present study detected signals for adalimumab and infliximab.

ILD has emerged as an adverse event associated with TNF‐α inhibitor use, and our results support the revision of the package insert by the Ministry of Health, Labour and Welfare. No signals were detected for certolizumab, ustekinumab, secukinumab, brodalumab, ixekizumab, guselkumab, and risankizumab, whereas ILD is listed as a serious adverse effect for certolizumab, ustekinumab, and ixekizumab, and several cases of a causal relationship with ILD onset have been reported [1]. Therefore, caution needs to be exercised when administering these drugs. After examining the potential involvement of primary diseases such as psoriasis, rheumatoid arthritis, and gastrointestinal inflammatory diseases in the development of ILD, no distinct correlations were observed between the primary diseases and the development of ILD (Table S3).

Progressive features of ILD disease, including idiopathic interstitial pneumonia, are exacerbation of respiratory symptoms, deterioration of respiratory function, progressive fibrosis on high‐resolution CT, rapid respiratory deterioration, or even death. In addition, pulmonary hypertension, gastroesophageal reflux, obstructive sleep apnea, obesity, cardiovascular disease, diabetes, and emphysema may coexist [26].

In this study, analysis of the occurrence of complications in patients who developed ILD as an adverse event upon administration of the TNF‐α inhibitors adalimumab, infliximab, and certolizumab showed complications such as tuberculosis and upper respiratory tract infection, acute respiratory failure, and dyspnea, respectively. Based on this analysis, it is highly likely that exacerbation of respiratory symptoms and deterioration of respiratory function are complications of ILD. Poor prognostic factors for lung injury caused by bleomycin and gefitinib (that cause ILD as an adverse event) and biologics include male sex, history of smoking, age over 70 years, history of lung disease, and renal impairment [27]. Patients with these predisposing factors are at risk of developing complications even with TNF‐α inhibitors such as adalimumab, infliximab, and certolizumab.

Adalimumab is associated with infectious respiratory diseases such as tuberculosis, and infliximab and certolizumab are associated with other respiratory diseases such as acute respiratory failure and dyspnea. The complications associated with these drugs are different, even though they have the same effect of inhibiting TNF‐α expression. The reason for the differences in the occurrence of respiratory diseases with different biologics may be that each drug acts through a different mechanism, such as suppressing the immune system's ability to protect against infection [1] or causing fibrosis of the lungs due to direct cellular damage [27].

When diagnosing drug‐induced ILD, it is important to ascertain the temporal relationship between the administration of the suspected drug and the onset of lung injury through a detailed interview and investigation. However, although drug‐loading tests are useful for identifying the causative drug, there are ethical issues involved, and no consensus has been reached on whether they should be performed [26]. Therefore, data on the temporal relationship between the administration of the suspected drug and the development of drug‐induced ILD are lacking.

Here, the median time from the start of treatment to the onset of ILD ranged from 82.0 to 320.5 days for TNF‐α inhibitors such as adalimumab, infliximab, and certolizumab, and from 90 to 206.5 days for IL inhibitors such as ustekinumab, secukinumab, ixekizumab, and guselkumab. There was little discrepancy between TNF‐α inhibitors and IL inhibitors in terms of the median time from the start of administration to the onset of ILD. Furthermore, there was a substantial difference in the median time from the start of treatment to the onset of ILD (82.0 days for certolizumab and 320.5 days for adalimumab). Therefore, it is imperative to monitor the timing of adverse events while considering the timing at which adverse events are most likely to occur. This study provides insights into the previously unknown temporal relationship between biologic drug administration and the onset of drug‐induced ILD. Our findings revealed that the timing of ILD onset varies with the biologic drug, ranging from 90 to 320.5 days. It is important to pay attention to the timing of adverse event onset when administering biologic agents.

The limitations of this study are that VigiBase relies on spontaneous reporting of adverse events and only provides comparisons among cases in which adverse events occur, without any information on the patient population not using the target drug. This renders it unfeasible to compare risks associated with different drugs. In addition, there may be potential bias present in the dataset from reported cases. Therefore, not all factors that could influence the results (such as concomitant medications) are reported. Missing data are an inherent limitation of the data sources used, and selection bias may arise when excluding reports with missing data. Not all national drug safety monitoring centers contributing to VigiBase perform Individual Case Safety Report causality assessments; therefore, missing data may be caused due to differences in national reporting systems [28, 29]. Additionally, it is important to acknowledge potential biases in the dataset such as over‐reporting of adverse events due to recent safety information updates or package insert revisions.

ILD has received considerable attention as an adverse event associated with biologics in clinical practice and has been included in package inserts. However, it was not previously known that the timing of ILD onset differs with each biologic agent and that there are differences in the classification of concomitant respiratory diseases, such as infections or respiratory failure. In this study, we investigated the effects of biologics on potential ILD complications. We also identified probable complications associated with each biologic in patients who developed ILD, along with the timeframe from the administration of the biologic to ILD onset. It is necessary to consider the predisposing factors to ILD when using biologic agents to treat psoriasis and rheumatism to ensure safe drug therapy as the pathogenesis and pathophysiology of ILD remain largely unknown [27], and treatment methods have not been established.

Based on our results, it is important to take into account the classification of respiratory diseases that are likely to be caused by each biologic agent. Drugs should be selected based on the patient's condition and actions taken in accordance with the characteristics of each drug, which will help to prevent the occurrence of ILD and concomitant complications. The study also revealed that the complications likely to occur with different biologics differ, but the mechanism of ILD occurrence and the reason for such differences among drugs, even though they all act in the same way to inhibit TNF‐α expression, are still unclear. Cytokines such as TNF‐α inhibitors and IL‐17 are associated with the development of ILD [7, 8], but which cytokines are involved in the development of complications and the underlying mechanisms remain unclear. Therefore, we believe that multifaceted studies, including this large‐scale database analysis, to elucidate the mechanisms underlying ILD development using human cells and mice, and clinical research to clarify the relationship with complications that are likely to occur in different patients, will lead to more effective and safer use of pharmaceutical products.

## Author Contributions

Ayu Minagi and Hideki Nawa wrote the manuscript. Ayu Minagi, Hideki Nawa, Mitsuhiro Goda, Takahiro Niimura, Koji Miyata, Hirofumi Hamano, Yoshito Zamami, and Keisuke Ishizawa designed the study. Ayu Minagi, Hideki Nawa, Mitsuhiro Goda, and Keisuke Ishizawa performed the study. Ayu Minagi, Hideki Nawa, Mitsuhiro Goda, Takahiro Niimura, Koji Miyata, Hirofumi Hamano, and Yoshito Zamami analyzed the data.

## Ethics Statement

This study was exempt from obtaining ethical approval and informed consent. The applicable regulations regarding ethics in this study are the “Ethical Regulations for Epidemiological Research” in Japan, https://www.mhlw.go.jp/general/seido/kousei/i‐kenkyu/ekigaku/0504sisin.html. Anonymized data were used in our analyses; therefore, informed consent was not required as per the “Ethical Regulations for Epidemiological Research,” which are the regulations stipulated by the Ministry of Health, Labour and Welfare of Japan. Data in VigiBase, the WHO global database of reported potential adverse effects of medicinal products, are developed and maintained by the Uppsala Monitoring Centre. The information originates from a variety of sources, and the probability that a suspected adverse effect is drug‐related is not the same in all cases. The information does not represent the opinion of the UMC or the World Health Organization.

## Conflicts of Interest

The authors declare no conflicts of interest.

## Supporting information


Data S1.

## Data Availability

The data that support the findings of this study are available in the Supporting Information of this article.
