# Supplementary material for: Evaluation of Interstitial Lung Disease Complications Caused by Biologic Agents Using a Spontaneous Adverse Drug Reaction Reporting Database
Source: Pharmacol Res Perspect. 2025 Feb 21;13(2):e70063. doi: 10.1002/prp2.70063 (PMC11845275; doi:10.1002/prp2.70063)
Supplement: Supplementary file 1 — Data S1. [file PRP2-13-e70063-s001.docx]

**Supplementary Table 1** Preferred terms (PTs) in interstitial lung disease (standardized MedDRA queries (SMQ): 20000042)

| CODE | PT | CODE | PT |
| --- | --- | --- | --- |
| 10066728 | Acute interstitial pneumonitis | 10022611 | Interstitial lung disease |
| 10073344 | Alveolar lung disease | 10086117 | Low lung compliance |
| 10001881 | Alveolar proteinosis | 10025102 | Lung infiltration |
| 10001889 | Alveolitis | 10081792 | Lung opacity |
| 10050343 | Alveolitis necrotizing | 10070831 | Necrotizing bronchiolitis |
| 10080701 | Autoimmune lung disease | 10029888 | Obliterative bronchiolitis |
| 10006448 | Bronchiolitis | 10084305 | Pleuroparenchymal fibroelastosis |
| 10083303 | Bronchiolitis obliterans syndrome | 10035742 | Pneumonitis |
| 10086041 | Chronic graft versus host disease in lung | 10085188 | Probable e-cigarette or vaping product use  associated lung injury |
| 10076515 | Combined pulmonary fibrosis and emphysema | 10036805 | Progressive massive fibrosis |
| 10085189 | Confirmed e-cigarette or vaping product use  associated lung injury | 10037383 | Pulmonary fibrosis |
| 10060902 | Diffuse alveolar damage | 10058824 | Pulmonary necrosis |
| 10014952 | Eosinophilia myalgia syndrome | 10061473 | Pulmonary radiation injury |
| 10078117 | Eosinophilic granulomatosis with polyangiitis | 10061924 | Pulmonary toxicity |
| 10014962 | Eosinophilic pneumonia | 10037457 | Pulmonary vasculitis |
| 10052832 | Eosinophilic pneumonia acute | 10037754 | Radiation alveolitis |
| 10052833 | Eosinophilic pneumonia chronic | 10085628 | Radiation bronchitis |
| 10081988 | Hypersensitivity pneumonitis | 10037758 | Radiation fibrosis - lung |
| 10078268 | Idiopathic interstitial pneumonia | 10037765 | Radiation pneumonitis |
| 10063725 | Idiopathic pneumonia syndrome | 10085517 | Rheumatoid arthritis-associated  interstitial lung disease |
| 10021240 | Idiopathic pulmonary fibrosis | 10080547 | Small airways disease |
| 10085352 | Immune-mediated lung disease | 10052235 | Transfusion-related acute lung injury |

PT= Preferred Term

**Supplementary Table 2** IC (95% CI) for ILD onset upon administration of biological agents

| Biological agent | Case/Total | IC (95% Cl) |
| --- | --- | --- |
| Adalimumab | 1632/654103 | 0. 24 (0.16–0.30) |
| Infliximab | 1159/190629 | 1.53 (1.43–1.60) |
| Certolizumab | 169/68450 | 0.23 (−0.03 to 0.41) |
| Ustekinumab | 98/66234 | −0.51 (−0.84 to −0.27) |
| Secukinumab | 90/113185 | −1.40 (−1.75 to −1.15) |
| Brodalumab | 1/2396 | −1.89 (−5.67 to −0.20) |
| Ixekizumab | 15/27597 | −1.92 (−2.79 to −1.32) |
| Guselkumab | 6/12367 | −2.03 (−3.44 to −1.12) |
| Risankizumab | 17/9653 | −0.25 (−1.07 to 0.32) |

A signal was present when the lower limit of the 95% confidence interval (census interval: CI) of the calculated IC was greater than 0. IC = information component.

**Supplementary Table 3** IC (95% CI) for primary disease and ILD onset

| Biological agent | Case/Total | IC (95% Cl) |
| --- | --- | --- |
| Psoriatic conditions |  |  |
| Adalimumab | 174/110385 | 0.32 (0.07–0.50) |
| Infliximab | 58/8961 | 2.31 (1.87–2.62) |
| Certolizumab | 8/6620 | −0.06 (−1.27 to 0.74) |
| Ustekinumab | 45/21183 | 0.74 (0.24–1.09) |
| Secukinumab | 45/56074 | −0.65 (−1.14 to −0.29) |
| Brodalumab | 1/1436 | −0.63 (−4.41 to 1.06) |
| Ixekizumab | 11/15431 | −0.80 (−1.82 to −0.10) |
| Guselkumab | 4/6174 | −0.88 (−2.65 to 0.20) |
| Risankizumab | 17/7390 | 0.83 (0.02–1.40) |
| Rheumatoid arthritis and associated conditions |  |  |
| Adalimumab | 871/189331 | −0.12 (−0.24 to −0.04) |
| Infliximab | 454/33146 | 1.45 (1.29–1.56) |
| Certolizumab | 118/25559 | −0.12 (−0.42 to 0.10) |
| Ustekinumab | 1/481 | −0.96 (−4.74 to 0.73) |
| Secukinumab | 1/913 | −1.76 (−5.54 to −0.07) |
| Brodalumab | 0/4 | N/A |
| Ixekizumab | 0/233 | N/A |
| Guselkumab | 0/48 | N/A |
| Risankizumab | 0/42 | N/A |
| Colitis (excl infective) |  |  |
| Adalimumab | 178/163866 | −1.06 (−1.31 to −0.88) |
| Infliximab | 183/70118 | 0.20 (−0.04 to 0.38) |
| Certolizumab | 13/13524 | −1.21 (−2.15 to −0.56) |
| Ustekinumab | 21/17845 | −0.93 (−1.66 to −0.42) |
| Secukinumab | 0/63 | N/A |
| Brodalumab | 0/2 | N/A |
| Ixekizumab | 0/15 | N/A |
| Guselkumab | 0/27 | N/A |
| Risankizumab | 0/71 | N/A |

A signal was present when the lower limit of the 95% confidence interval (census interval: CI) of the calculated IC was greater than 0. IC = information component. N/A = not applicable
